# Effect of Waterlogging Duration at Different Growth Stages on the Growth, Yield and Quality of Cotton

**DOI:** 10.1371/journal.pone.0169029

**Published:** 2017-01-03

**Authors:** Xiaosen Wang, Zhong Deng, Wenzheng Zhang, Zhaojiang Meng, Xiao Chang, Mouchao Lv

**Affiliations:** 1 Farmland Irrigation Research Institute, Chinese Academy of Agricultural Sciences, Muye District, Xinxiang City, Henan province, China; 2 Key Laboratory of Water-Saving Agriculture of Henan Province, Muye District, Xinxiang City, Henan province, China; 3 Center for Efficient Irrigation Engineering and Technology Research, CAAS, Muye district, Xinxiang City, Henan province, China; Chinese Academy of Agricultural Sciences Cotton Research Institute, CHINA

## Abstract

In this study, a soil culture experiment was set up in barrels to investigate the effect of waterlogging duration at different growth stages on the growth, yield, and quality of cotton in the Huang-Huai Region of China during summer. The experiment was conducted at four growth stages of cotton (seedling, squaring, flowering, and boll opening), and the waterlogging duration at each stage was set to five levels (2, 4, 6, 8, and 10 d) and the waterlogging depth was 5cm. Twenty different treatment combinations were established, and one group without waterlogging throughout the whole growth period was used as the control (CK). The results showed that the waterlogging treatments at the different growth stages reduced the morphological and yield parameters of the cotton plants as well as the physiological parameters of the cotton leaves, and the extent of the reduction in these parameters increased with the extension of the waterlogging duration. The effect of waterlogging at different growth stages on the cotton decreased in the order of the flowering, squaring, seedling, and boll-opening stages, and the highest yield reduction rates for the four stages were 38.8%, 27.9%, 18.3%, and 7.6% respectively. Additionally, waterlogging decreased the quality parameters of cotton such as the upper-half mean length, uniformity index, micronaire value, elongation, yellowness, and lint percentage at the squaring, flowering, and boll-opening stages. Furthermore, at the seedling stage waterlogging for no more than 6 d allowed the morphological and yield parameters to recover in the boll-opening stage upon timely drainage, and these parameters showed no significant decreases compared with the CK level. The critical duration of waterlogging at the squaring stage was 4 d. However, at the flowering stage, even 2 d of waterlogging could lead to the stagnation of morphological development and prevent the recovery of the cotton yield to the CK level. Therefore, when waterlogging disasters occur in cotton fields, the implementation of appropriate surface and subsurface drainage schemes for the different growth stages is needed as soon as possible to mitigate the damage.

## Introduction

Cotton is planted extensively following the wheat harvest in the Huang-Huai Region of China. The growth period of cotton generally lasts from mid-June to early October, which coincides with the local rainy season. Thus, waterlogging disasters often occur due to heavy rains, leading to yield losses and even resulting in total crop failure of the cotton, causing a substantial loss to local agricultural development and farmers’ incomes.

Cotton is a crop with poor tolerance to waterlogging. The number of days and depth of waterlogging, as well as the growth stage during waterlogging, can affect the growth and development of the cotton and its physiological metabolism, yield, and quality [1–6]. For example, it has been shown that waterlogging stress at the squaring and flowering stages significantly inhibits the morphological development of cotton, and leaf area growth is subject to the greatest inhibitory effect of waterlogging stress, followed by stem diameter and plant height[7]. Waterlogging reduces the chlorophyll content as well as the leaf photosynthetic rate of cotton, and alters the characteristics of biomass accumulation in the cotton bolls [8–10]. In terms of cotton yield, higher fruit position, a lower number of total fruit nodes, decreased boll number and weight, and increased boll abscission and rot rates occur with an increasing number of days of waterlogging, and the longer the waterlogging duration is, the greater the extent of yield reduction[11–14]. As for the cotton fiber quality, some reports say that waterlogging reduces the fiber length, specific strength, and uniformity as well as the lint percentage, seed index, and lint index, but some studies have also shown that waterlogging exhibits no effect on the cotton quality[11]. For post-disaster recovery, the roots of the cotton recover faster than the crown after waterlogging, and the reduction of oxidized roots is faster at the squaring stage compared with the flowering stage[15].

Taking into consideration the existing results obtained in China and other countries, in-depth researches have been conducted to reveal the mechanism for the effect of waterlogging disasters on cotton and they are very merit to guide the management of the waterlogging cotton. However, they focused mainly on a specific growth stage of cotton. Systematic studies are lacking on cotton with various waterlogging durations at the whloe growth stage, and few of them figure out the critical durations of waterlogging under different growth stages beyond which the cotton yield will decreased dramatically, which is important to make farmers take timely measures of drainage and appropriate ways of remedial measures to waterlogging cotton at different growth stages. In addition, there had existed some contradictory results of research which need to be clarified through elaborate experiments, such as the effect of waterlogging on the quality of cotton.

Therefore, in this study, four growth stages of cotton, i.e., seedling, squaring, flowering, and boll opening, were chosen for this experiment. We systematically studied the effect of various waterlogging durations at different growth stages on the growth, yield, and quality of the cotton. I hope these results would provide valuable reference for watterlogging disaster alleviating to the cotton planted not only in China but also around the world.

## Materials and Methods

### 2.1 Study site and plant materials

The experiment was conducted under a canopy at the Farmland Irrigation Research Institute of the Chinese Academy of Agricultural Sciences (CAAS) from June to September 2014. The experimental site is located at the coordinates of 35°19' N and 113°53' E in Xinxiang City, Henan Province, China. The average altitude is 73.2 m, and the annual average temperature is 14.1°C. The frost-free period ranges from 213 to 241 d, and the sunshine period varies between 2,200 and 2,400 h.

The experiment was set up in barrels using the soil culture method, and the soil was a sandy loam, with a bulk density of 1.38 g·cm^-3^ and a field water capacity (FC) of 24% (the mass percentage of water accounting for dry soil mass). The barrels were cylindrical and made up of galvanized iron sheet, and each comprised an inner barrel 40 cm in diameter and an outer barrel 41 cm in diameter. Both the inner and outer barrels were 60 cm in depth and contained a bottom. The inner barrel was placed inside the outer barrel and then buried in the ground, with the top edge higher than the ground level about 5cm. Each inner barrel was filled with 28.23 kg of dry soil, and 11 g of ternary compound fertilizer (N, P_2_O_5_, and K_2_O: 17% each) was applied as the base fertilizer and mixed thoroughly with the soil. A 5-cm-diameter polypropylene (PP) pipe was set up at two sides of the barrel, and the length of the pipe tube was slightly greater than the depth of the barrel. The PP pipe was drilled with small holes before use. Water was added into the PP pipe, and it passed through the small holes, thereby providing uniform irrigation. Four 1-cm-diameter holes were drilled evenly around the bottom of the inner barrel and plugged with corks during the waterlogging experimental period. At the end of the waterlogging experiment, the corks were removed to drain the water in the barrel. The water depth in all barrel sets was kept at approximately 5 cm during the waterlogging experiment.

The cultivar of Cotton is *Zhongmiansuo* 50 which is a transgenic insect resistant, early maturing variety bred by Institute of Cotton Research of CAAS and its whole growth period is about 110d in summer sowing condition at Huang-Huai Region of China. Cottons were cultured in the medium before transplanting, on June 15 (trefoil stage), robust seedlings with equivalent growth vigor were transplanted into these barrels, one plant per barrel. At the flowering stage of cotton, each barrel was top dressed with 2 g urea (46% N) and 2 g potassium sulfate (46% K_2_O) respectively.

### 2.2 Experimental treatment and design

The experiment was performed using a randomized block design with two factors, i.e., growth stage and waterlogging duration. The growth stage was divided into the seedling, squaring, flowering, and boll-opening stages. In each stage, the waterlogging duration was set to five levels, i.e., 2, 4, 6, 8, and 10 d. The number of each treatment is shown in Table 1. Waterlogging experiments at different growth stages began on June 30 (15d after transplanting, AT) for seedling stage, July 15 (30d AT) for squaring stage, August 12 (58d AT) for flowering stage, and September 5 (82d AT) for boll-opening stage respectively. The 10-d waterlogging treatment was started at the beginning of the waterlogging experiment, the 8-d waterlogging treatment was started 2 d later, and so on until the 2-d waterlogging treatment was conducted. This approach guaranteed the same end time for all experimental levels and therefore facilitated subsequent measurements of the morphological and physiological parameters. Twenty different treatment combinations were performed with 3 replicates per treatment. Additionally, a nonwaterlogging treatment was included as the control (CK), with 10 replicates. In total, 70 barrels were used in this experiment. The cultivation practices were performed according to the standard for high-yield cotton in the field.

**Table 1:** Abbreviations of waterlogging treatments at the different growth stages of cotton

Waterlogging duration(d)	Growth stage			
	Seedling	Squaring	Flowering	Boll opening
2	S2	B2	F2	T2
4	S4	B4	F4	T4
6	S6	B6	F6	T6
8	S8	B8	F8	T8
10	S10	B10	F10	T10

### 2.3 Measurement items and analytical methods

Soil moisture content was determined by weighing the inner barrel set at 8:00 am every day.The lower limit of irrigation was set to 75% FC for the CK and other cotton treatments in the nonwaterlogging period. These treatments were irrigated to FC when the soil moisture content dropped to 75% FC. Water was added using a measuring cup to quantify the volume. For cotton treatments in the waterlogging period, the water depth was kept at 5 cm in all barrel sets. At the ending of each waterlogging duration treatment, the water at the inner barrels was drained through the holes at the bottom of barrels immediately.

The effect of different waterlogging durations on the plant height and leaf area of cotton was investigated by using a straightedge and a steel ruler and the leaf area was estimated using the following equation:

S=L×W×0.75

where S is the leaf area (cm^2^), L is the leaf length (cm) and W is the leaf width (cm). The investigation was conducted on July 10, July 25, August 22, and September 15 for waterlogging at the seedling, squaring, flowering, and boll-opening stages, respectively, which were the next day of the end of each waterlogging experiment. On September 17–18, the plant height and leaf area were remeasured at the boll-opening stage of the cotton for the waterlogging treatments at the seedling, squaring, and flowering stages.

The leaves on the inverse fourth main stems were selected from the cotton plants, and the chlorophyll content and the photosynthetic rate were measured using a SPAD-502 chlorophyll meter (made in Japan Konica Minolta Ltd) and Li-6400 photosynthesis system (made in U.S.A Li-Cor Ltd) under 1000 μmol·m^-2^·s^-1^ light intensity at about 10:00 a.m, with three replicates per treatment. The measurements were taken at the squaring and flowering stages of the cotton at the same time as those taken for the plant height and leaf area of each stage.

The number of fruit branches, fruit nodes, and bolls and the weight of a single boll of cotton were surveyed for the waterlogging treatments and CK group prior to harvest. The shoot and root biomass of the cotton was collected after harvest. The samples were deactivated at 105°C for 0.5 h and then dried at 80°C in an oven to constant weight.

At the harvest stage, the bolls of each plant were harvested separately. The seed cotton yield and lint percentage were determined. Eight quality parameters of the cotton were determined at the Supervision, Inspection, and Test Center of Cotton Quality, Ministry of Agriculture of China. These parameters included the upper-half mean length, uniformity index, micronaire value, elongation, reflectance, yellowness, spinning consistence index, and specific breaking strength.

### 2.4 Statistics analysis

The data presented in this paper are means (±SD) of three replications of the same treatment.Their differences were analyzed by ANOVA (SAS Institute, Ver8.1) with Duncan’s multiple range tests at P = 5% level. All of the figures in this paper were created with EXCEL 2007 (Microsoft Office, Ver 2007).

## Results

### 3.1 Effects of waterlogging at different growth stages on the plant heights and leaf areas of cotton

**Fig 1:**
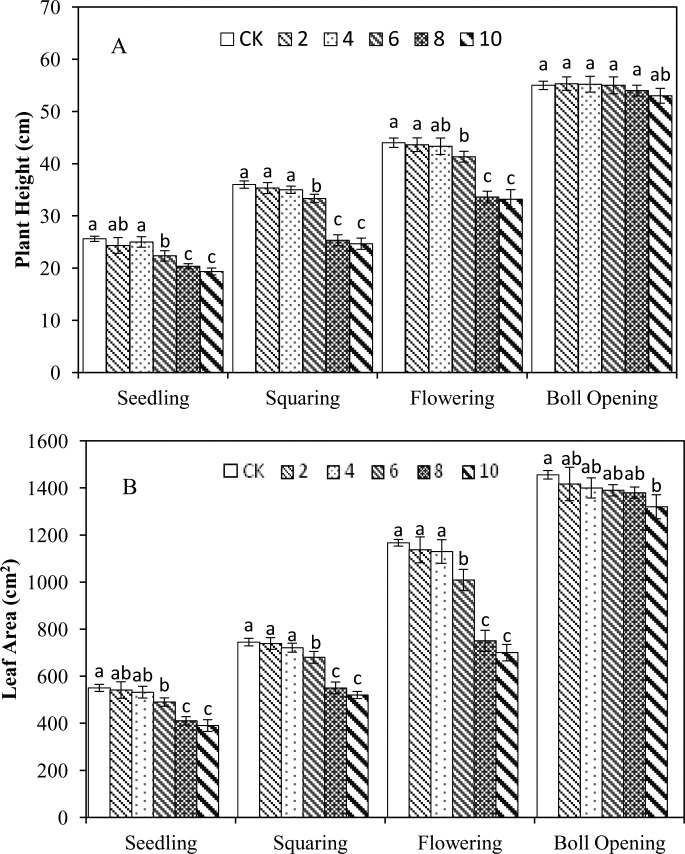
**The immediate effects of various waterlogging durations at the different growth stages on the plant heights (A) and leaf areas (B) of cotton.** The statistical analysis is one way ANOVA, and different letters on bars within the same growth stage are significantly different at P = 0.05 probability level.

The immediate effects of various waterlogging durations on the plant heights and leaf areas of cotton at the different growth stages are presented in Fig 1A and 1B. The results showed that the waterlogging treatment at the different growth stages affected the morphological development of the cotton, and the longer the waterlogging duration was, the slower the growth of the plant height and leaf area. Furthermore, the plant heights and leaf areas showed significant differences after 6 ds of waterlogging at the seedling, squaring, and flowering stages compared with the CK level (P <0.05), and these two parameters were reduced slightly but not significantly within 4d of waterlogging duration. The plant height showed the largest reduction of 31.50% with 10 d of waterlogging duration at the squaring stage, whereas the leaf area exhibited the largest reduction of 40.02% with 10 d of waterlogging duration at the flowering stage. Waterlogging at the boll-opening stage had no significant effect on the plant height; however, the leaf area became significantly smaller than the CK value after 10 d of waterlogging (P <0.05).

**Fig 2:**
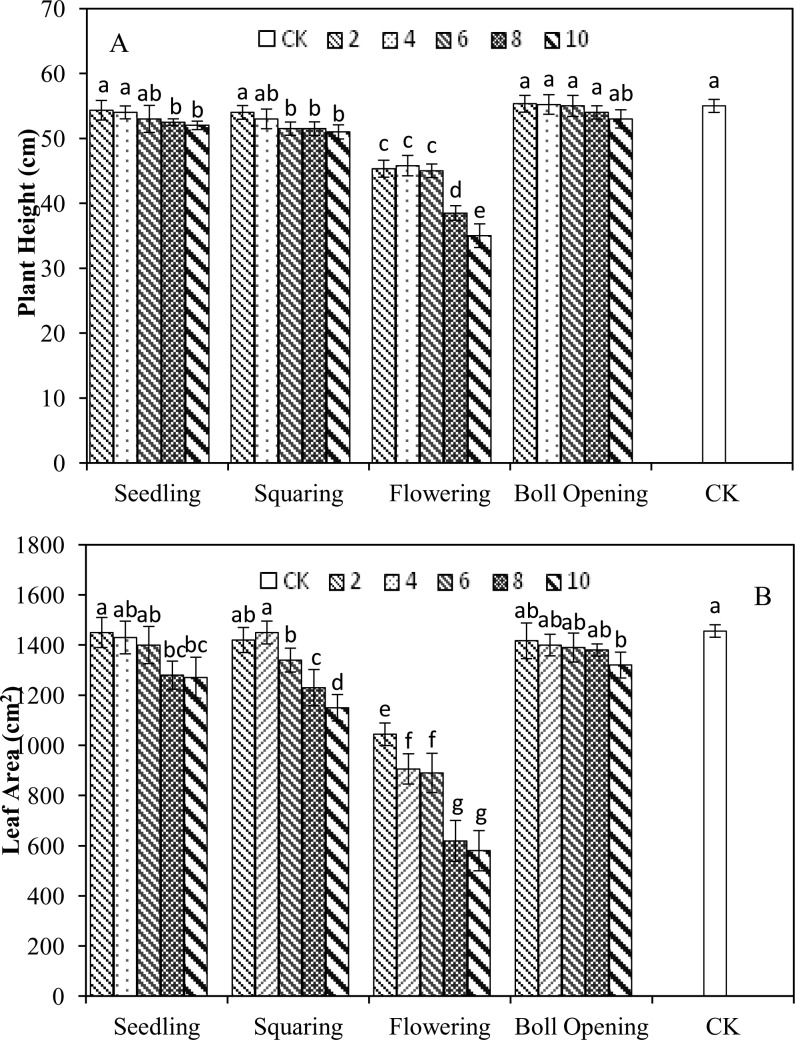
**The final effects of various waterlogging durations at the different growth stages on the plant heights (A) and leaf areas (B) of cotton.** The statistical analysis is two way ANOVA, and Different letters on bars are significantly different at P = 0.05 probability level.

The final effects of various waterlogging durations on the plant heights and leaf areas of cotton at the different growth stages are presented in Fig 2A and 2B. The plant heights and leaf areas of cotton at the boll-opening stage were compared among the whole waterlogging treatments in this experiment. For these treatments waterlogged at the seedling stage, both the plant heights and the leaf areas of S2, S4 and S6 treatments were able to recover at their boll-opening stage, showing no significant differences with CK level. However, these two parameters of S8 and S10 treatments remained significantly smaller than CK level (P <0.05). For these treatments waterlogged at the squaring stage, the plant heights and leaf areas of B2 and B4 treatments could recover at their boll-opening stage too, showing no significant differences with the CK level. But, these two parameters of B6, B8, and B10 treatments were significantly smaller than the CK level (P <0.05). As for these treatments waterlogged at the flowering stage, the plant heights and leaf areas of all waterlogging treatments were still significantly lower than the CK levels (P <0.05) at their boll-opening stage. The plant heights and leaf areas of all waterlogging treatments in this experiment ranked as T2>T4>CK = T6>S2>S4 = B2 = T8>S6 = B4 = T10>S8>S10>B6 = B8>B10>F4>F2>F6>F8>F10 and CK>S2 = B4>S4>B2>T2>S6 = T4>T6>T8>B6>T10>S8>S10>B8>B10>F2>F4>F6>F8>F10 respectively. From the results above mentioned, one conclusion can be made that the morphological development of the cotton waterlogged at the different growth stages exhibited a certain compensatory growth ability, but this ability varied with the growth stages and waterlogging durations.

### 3.2 Effects of waterlogging at different growth stages on the chlorophyll content (SPAD value) and photosynthetic (Pn) rate of cotton

**Fig 3:**
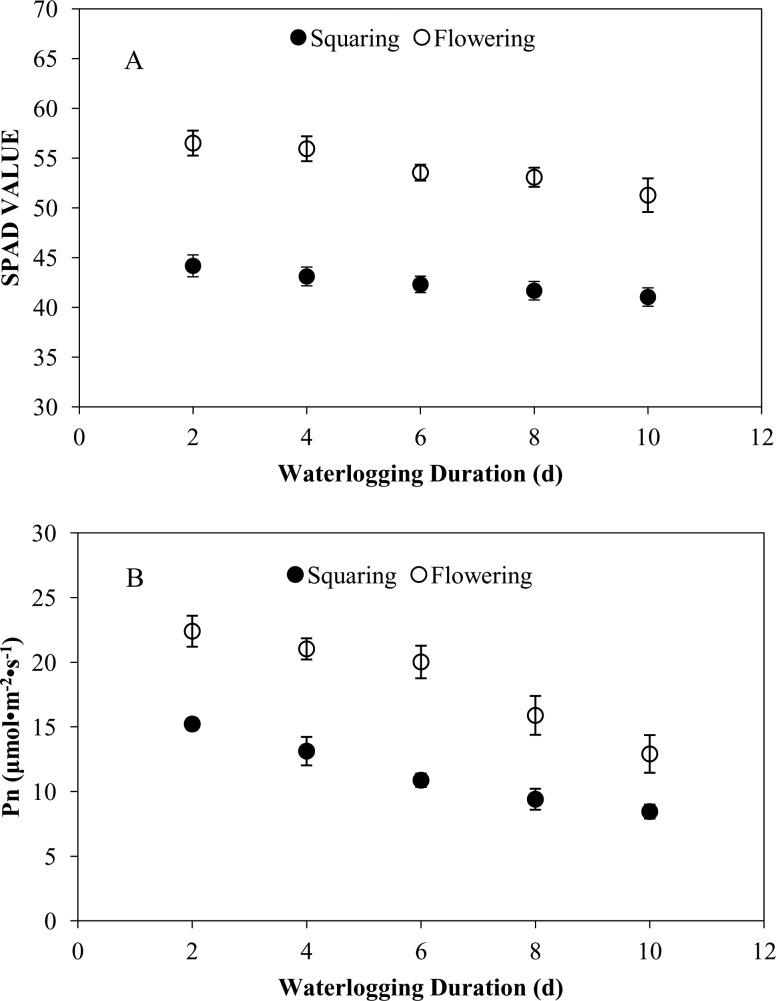
**The chlorophyll content (SPAD VALUE) (A) and photosynthetic rate (B) of the cotton with the waterlogging treatments at the squaring and flowering stages.** The statistical analysis is one way ANOVA.

The chlorophyll content and photosynthetic rate of the cotton with the waterlogging treatments at the squaring and flowering stages are presented in Fig 3A and 3B. After waterlogging treatment at these two stages, both parameters decreased with an increase in the waterlogging durations, and the values were significantly lower than the CK after 4 d of waterlogging (P <0.05). Moreover, the values of chlorophyll content and photosynthetic rate at the flowering stage were higher than those at the squaring stage.

### 3.3 Effects of waterlogging at different growth stages on the yield and dry matter components of cotton

The effects of the waterlogging treatments at the different growth stages on the yield and dry matter components of cotton are shown in Table 2. The numbers of fruit branches and nodes decreased with the increase of waterlogging duration at the different growth stages. Compared with the CK, the waterlogging treatments at the flowering stage most significantly reduced the numbers of fruit branches and nodes, followed by those at the squaring stage, and the waterlogging treatments at the seedling stage came third. However, the waterlogging treatments at the boll-opening stage almost had no effect on the numbers of fruit branches and nodes. This indicated that the flowering and squaring stages were the critical growth stages of cotton, at which waterlogging disasters even 2d could seriously affect the subsequent development of fruit branches. At the seedling stage, short-term waterlogging for less than 4 d did not significantly affect the subsequent growth of the fruit branches of the cotton upon timely drainage, however, more than 6 d of waterlogging still significantly reduced the number of fruit branches (P <0.05). At the boll-opening stage, the cotton plants had finished its vegetative growth, and waterlogging at this stage had almost no effect on the numbers of fruit branches and nodes.

**Table 2:** The effects of the waterlogging treatments at the different growth stages on the fruit branch, fruit branch node, boll number, boll weight, seed cotton yield, yield reduction rate, dry matter and RSR of cotton.

Waterlogging	Waterlogging	Fruit branch		Fruit branch		Boll number		Boll		Seed cotton		Yield reduction	Aboveground		Root dry		RSR
stages	duration (d)	number		node number				weight (g)		yield (g)		rate (%)	dry matter (g)		matter (g)		
Seedling	2	15.0	ab	28.7	ab	11.0	ab	4.5	ab	41.8	ab	0.5	23.8	ab	6.3	ab	0.26
	4	14.5	ab	28.0	ab	10.6	ab	4.6	a	41.8	ab	0.5	22.7	bc	5.6	cd	0.25
	6	14.0	bc	26.0	bc	10.0	bc	4.0	bc	40.0	bc	4.8	22.5	bc	5.3	de	0.24
	8	11.5	ef	25.0	bc	8.5	cd	4.0	bc	36.5	cd	13.1	20.6	cd	4.6	ef	0.22
	10	10.0	gh	23.0	cd	8.0	cd	3.8	cd	34.3	cd	18.3	19.1	cd	4.1	fg	0.21
Squaring	2	13.0	cd	26.5	bc	10.3	ab	4.5	ab	40.2	ab	4.3	23.2	ab	6.0	bc	0.26
	4	12.7	cd	26.3	bc	10.0	bc	4.4	ab	40.0	bc	4.8	21.2	bc	5.5	cd	0.26
	6	12.3	de	25.8	bc	9.9	bc	4.0	bc	38.5	**bc**	8.3	21.7	**bc**	5.0	**de**	0.23
	8	10.5	fg	23.7	cd	8.8	cd	3.9	bc	33.5	cd	20.2	19.4	cd	4.7	ef	0.24
	10	9.8	h	22.5	de	7.5	de	3.6	cd	30.3	e	27.9	18.6	cd	4.3	fg	0.23
Flowering	2	12.0	de	25.5	bc	8.3	cd	4.0	bc	36.0	cd	14.3	20.5	cd	4.5	ef	0.22
	4	11.5	ef	24.5	cd	7.3	de	3.9	bc	32.3	de	23.1	16.2	d	4.2	fg	0.26
	6	10.5	fg	24.0	cd	7.6	de	3.7	cd	30.8	de	26.7	16.0	d	4.0	fg	0.25
	8	8.5	i	19.5	ef	5.5	ef	3.4	de	26.5	f	36.9	12.6	e	3.0	h	0.24
	10	8.3	i	19.0	f	4.0	f	3.2	e	25.7	f	38.8	12.4	e	2.6	h	0.21
Boll-opening	2	15.5	a	29.0	a	11.2	a	4.6	a	42.0	a	0.0	26.4	a	6.5	a	0.25
	4	15.0	ab	28.7	ab	11.0	ab	4.5	ab	41.8	ab	0.5	24.6	ab	6.4	ab	0.26
	6	15.0	ab	28.7	ab	10.0	bc	4.4	ab	40.0	bc	4.8	23.8	ab	6.3	ab	0.26
	8	14.5	ab	28.5	ab	9.7	bc	4.0	bc	40.0	bc	4.8	24.8	ab	5.4	de	0.22
	10	14.5	ab	28.5	ab	9.5	bc	4.0	bc	38.8	bc	7.6	20.8	cd	4.4	fg	0.21
CK		14.5	ab	28.5	ab	11.2	a	4.6	a	42.0	a	0.0	24.1	ab	6.5	a	0.27

The statistical analysis is two way ANOVA, and values followed by different letters within the same row are significantly different at P = 0.05 probability level.

The effects of the waterlogging treatments at the different growth stages on the boll number and single-boll weight of the cotton are shown in Table 2. Both parameters were decreased with the increase of waterlogging duration at the different growth stages. In terms of the boll number, the values became significantly lower than the CK level after 6 d of waterlogging at the seedling and boll-opening stages (P <0.05). A significant reduction was observed in the boll number after 4 d of waterlogging at the squaring stage (P <0.05), and even 2 d of waterlogging at the flowering stage could significantly reduce the boll number compared with the CK level (P <0.05). In terms of the single boll weight, the values became significantly lower than the CK level after 6 d of waterlogging at the seedling and squaring stages (P <0.05). This parameter was significantly reduced after only 2 d of waterlogging at the flowering stage (P <0.05), but up to 8 d of waterlogging at the boll-opening stage (P <0.05) compared with the CK level. For the waterlogging treatments and the CK, the boll number and single boll weight ranked as CK = T2>S2 = T4>S4>B2>S6 = B4 = T6>B6>T8>T10>B8>S8>F2>S10>F6>B10>F4>F8>F10 and CK = T2 = S4>S2 = B2 = T4>B4 = T6>S6 = S8 = B6 = F2 = T8 = T10>B8 = F4>S10>F6>B10>F8>F10 respectively.

The boll number and single boll weight are two important parameters used to evaluate cotton yield. A reduction in these two parameters inevitably leads to a decline in the seed cotton yield and crop failure. Table 2 shows the seed cotton yield for the waterlogging treatments and the CK. The yield became significantly lower than the CK levels after 6 d of waterlogging at the seedling and boll-opening stages (P <0.05), 4 d of waterlogging at the squaring stage (P <0.05), and 2 d of waterlogging at the flowering stage (P <0.05). The highest yield reduction rates of the cotton were 18.3%, 27.9%, 38.8%, and 7.6% for the waterlogging treatments at the seedling, squaring, flowering, and boll-opening stages respectively. For the waterlogging treatments and the CK, the cotton yield ranked as CK = T2>S2 = S4 = T4>B2>S6 = B4 = T6 = T8>T10>B6>S8>F2>S10>B8>F4>F6>B10>F8>F10.

Waterlogging not only causes yield reduction but also significantly affects the aboveground and belowground dry matter accumulation in cotton. As shown in Table 2, the weights of aboveground dry matters including stems, leaves and hulls, and the root dry matters decreased with the increase of waterlogging duration in the various treatments. Compared with the CK, the waterlogging treatments at the flowering stage reduced the dry matter weight most, followed by those at the squaring stage, and the treatments at the seedling stage came third. The least effect of waterlogging on the dry matter weight was found at the boll-opening stage. Moreover, the root-to-shoot ratio (RSR) of the cotton decreased with the increase of waterlogging durations compared to the CK.

### 3.4 Effects of waterlogging at different growth stages on the quality parameters of cotton

The effects of the waterlogging treatments at the different growth stages on the quality parameters of cotton are presented in Table 3. With the increase of waterlogging duration at the squaring, flowering, and boll-opening stages, the upper-half mean length, uniformity index, micronaire value, elongation, yellowness, and lint percentage gradually decreased, whereas the reflectance, spinning consistence index, and specific breaking strength gradually increased. Among the four growth stages, the effects of waterlogging on these quality parameters was greatest at the flowering stage and then the squaring stage, and the boll openning stage came third, least at the seedling stage.

**Table 3:** The effect of the waterlogging treatments at the different growth stages on the quality parameters of the cotton.

Waterlogging	Waterlogging	Upper-half		Uniformity		Micronaire		Elongation		Reflectance		Yellowness		Spinning		Specific		Lint	
stages	duration	mean length		index		value		percentage		percentage				consistence		breaking		percentage	
	(d)	(mm)		(%)				(%)		(%)				index		strength		(%)	
																(cN/tex)			
Seedling	2	27.40	ab	82.50	ab	4.55	cd	6.40	bc	74.20	c	8.30	ab	118.00	ab	27.25	ab	43	bc
	4	27.61	ab	82.90	ab	5.22	ab	6.90	a	75.10	bc	8.30	ab	120.00	ab	27.30	ab	42	bc
	6	28.15	ab	83.80	a	5.30	ab	7.00	a	75.40	ab	8.20	ab	124.00	a	27.50	ab	41	bc
	8	28.39	a	83.40	ab	5.34	ab	7.00	a	75.70	ab	7.70	bc	120.00	ab	27.93	ab	41	bc
	10	27.36	ab	83.00	ab	4.80	bc	6.90	a	76.01	a	7.80	bc	111.00	ab	27.90	ab	39	c
Squaring	2	27.43	ab	82.50	ab	5.29	ab	6.28	bc	74.50	bc	8.60	ab	107.00	bc	27.15	ab	44	ab
	4	27.33	ab	82.30	ab	4.78	bc	6.30	bc	75.20	ab	8.70	a	112.00	ab	28.22	ab	44	ab
	6	27.23	ab	82.10	ab	4.56	cd	6.30	bc	75.40	ab	8.60	ab	116.00	ab	28.28	ab	44	ab
	8	27.20	ab	81.90	bc	4.34	cd	6.30	bc	75.50	ab	7.90	ab	116.00	ab	28.32	a	43	bc
	10	27.12	ab	81.70	bc	4.18	d	5.70	e	75.90	ab	7.30	c	118.00	ab	28.32	a	41	bc
Flowering	2	26.52	bc	82.30	ab	5.41	ab	6.15	cd	74.50	bc	8.30	ab	92.00	c	25.97	c	46	a
	4	26.18	bc	81.80	bc	5.35	ab	6.15	cd	75.10	bc	8.30	ab	95.00	c	26.24	bc	46	a
	6	25.55	cd	81.50	bc	5.11	bc	6.10	cd	75.50	ab	7.90	ab	100.00	bc	26.66	bc	46	a
	8	25.08	cd	81.00	bc	5.24	ab	6.00	cd	75.60	ab	7.90	ab	113.00	ab	27.83	ab	45	ab
	10	24.84	d	80.90	cd	4.76	bc	6.00	cd	75.70	ab	7.50	bc	117.00	ab	27.83	ab	43	bc
Boll-opening	2	26.07	bc	82.20	ab	5.42	a	6.40	bc	74.30	bc	8.40	ab	97.00	c	26.46	bc	45	ab
	4	25.95	cd	81.80	bc	5.24	ab	6.40	bc	74.50	bc	8.30	ab	97.00	c	26.55	bc	46	a
	6	25.90	cd	81.40	bc	5.03	bc	6.30	bc	74.70	bc	8.10	ab	98.00	c	26.75	bc	44	ab
	8	25.29	cd	80.80	cd	4.99	bc	6.10	cd	75.20	ab	8.10	ab	100.00	bc	27.12	ab	44	ab
	10	25.08	cd	80.20	d	4.89	bc	6.00	de	75.80	ab	8.10	ab	109.00	bc	27.73	ab	45	ab
CK		27.48	ab	82.90	ab	4.48	cd	6.20	bc	74.30	bc	8.20	ab	117.00	ab	27.34	ab	46	a

The statistical analysis is two way ANOVA, and values followed by different letters within the same row are significantly different at P = 0.05 probability level.

## Discussion

According to some references, Waterlogging reduces the oxygen diffusion rate through soil and leads to O_2_ depletion, which inhibit the respiration of plant root, energy generation and nutrient acquisition and induce oxidative damage to cotton root tissues [6,10,16]. These belowground effects affect the shoots, interfering with canopy development [17]. In this study,waterlogging reduced the morphological and yield parameters of cotton as well, and the longer the waterlogging duration was, the greater the reduction in these parameters. With regard to the growth stage of cotton, the effect of waterlogging ranked as flowering stage > squaring stage > seedling stage > boll-opening stage. The flowering stage is a critical growth stage for the vegetative and reproductive growth of cotton, thus waterlogging stress at this stage can severely restrict the morphological development and yield formation of the cotton. However, At the boll-opening stage cotton plants have generally finished vegetative and reproductive growth, and waterlogging stress at this stage has little effect on the morphology and yield of the cotton. Morover, some experts noted that waterlogging inhibited cotton absorption of nourishment, nutrient concentrations in cotton leaves were relatively more sensitive to waterlogging during peak flowering compared with late reproductive stages [18–20]. In addition, the flowering stage of cotton occured in the hottest period of the year in this study, and the highest temperature in one day approched 36°C(centigrade degree), and the high temperature after waterlogging might increase the extent of yield reduction [21]. The possible reason was interpreted by Najeeb (2015) that the O_2_ depletion would be exacerbated by high temperatures, which accelerated respiratory activity of cotton root.

Through elaborate surveys of cotton bolls in this experiment, the yield reduction of cotton was mainly attributable to the decreases in the boll number and single boll weight, and the boll number decreased due to the increases in boll abscission and rot rates. Some literatures noted that waterlogging led to ethylene accumulation, which accelerated activity of an abscission layer in the peduncle, causing square and boll abscission and overall lint yield reduction in cotton [13,22–23].

According to the results of this experiment, the photosynthetic rate and chlorophyll content of cotton decreased dramatically due to long-term waterlogging. This is consistent with the conclusions of previous studies that waterlogging may induce stomatal closure, thereby decreasing transpiration and photosynthesis of plant [8,17]. Chlorophyll plays a central role in light absorption during the photosynthetic process. The reduction in the chlorophyll content could lower the efficiency of cotton to convert light energy into chemical energy, thereby suppressing the photosynthetic rate, reducing the total amount of organic synthesis, and ultimately leading to a reduction in the biomass of cotton plants. Furthermore, some experts declared that early reduction in photosynthesis is regulated by internal damage to photosystem II (PSII) and ultimately limits light interception in cotton [9,24–26].

Previous researches indicate that cotton plants possess complete mechanisms for protection and adaptation against waterlogging. When subjected to waterlogging stress, cotton plants can trigger the mechanisms of escape, static adaptation, and regeneration-compensation to adapt to waterlogging stress and mitigate waterlogging damage[26–27]. Through the waterlogging experiment at the whole growth stage of cotton, we found that the cotton plants exhibited a strong regeneration-compensation capacity after waterlogging at the seedling stage. With a waterlogging duration of no more than 6 d at seedling stage, although waterlogging posed temporarily effect on the morphology of the cotton plants, the relevant parameters could recover through regeneration-compensation and showed no significant differences compared with the CK level at the boll-opening stage, and no significant decreases in the boll number, single boll weight, and seed cotton yield were observed. However, with the waterlogging duration of more than 6 d, the mechanism of regeneration-compensation could not completely compensate for the harm of waterlogging to cotton growth and development, and the morphological and yield parameters were significantly lower than the CK level. Moreover, the mechanism of growth compensation in cotton gradually became less effective with the progression of the growth stages. At the squaring stage, the maximum duration of waterlogging that avoided significant yield reduction was 4 d, and more than 4 d of waterlogging still significantly reduced the morphological and yield parameters. At the flowering stage, even 2 d of waterlogging stress could prevent the recovery of morphological and yield parameters to CK levels. Therefore, some experts said that, after waterlogging treatment, new leaves recover more easily than mature functional leaves, and the leaves have higher recovery capability after the elimination of waterlogging stress in the early stage compared with the late stages [28].

## Conclusions

Waterlogging treatments at different growth stages of cotton reduced the morphological parameters and yield components as well as the chlorophyll content and photosynthetic rate in this crop. A longer waterlogging duration caused a greater reduction in the above parameters. Cotton plants showed the highest sensitivity to waterlogging at the flowering stage, followed by the squaring stage, the seedling stage came third, and the boll-opening stage was last. Additionally, waterlogging reduced the quality parameters of cotton, e.g., the upper-half mean length, uniformity index, micronaire value, elongation, yellowness, and lint percentage at the squaring, flowering, and boll-opening stages. At the seedling stage, no more than 6 d of waterlogging allowed the morphological and yield parameters of cotton recover to the CK level in the boll-opening stage upon timely drainage. At the squaring stage, the critical waterlogging duration was 4 d. However, at the flowering stage, even 2 d of waterlogging would cause stagnation of morphogenesis and prevent the yield recovery to the CK level. Therefore, the management of cotton fields in the Huang-Huai Region must be strengthened during the summer season. When floods occur, surface and subsurface drainage should be implemented to control the groundwater level and mitigate the economic losses of cotton to least level.

## Supporting Information

S1 File Basic datas of Figs 1–3. (XLSX)
